# Computed tomography pericoronary adipose tissue density predicts coronary allograft vasculopathy and adverse clinical outcomes after cardiac transplantation

**DOI:** 10.1093/ehjci/jeae069

**Published:** 2024-03-17

**Authors:** Christopher Wall, Jonathan Weir-McCall, Katharine Tweed, Stephen P Hoole, Deepa Gopalan, Yuan Huang, Andrej Corovic, Marta Peverelli, Damini Dey, Martin R Bennett, James H F Rudd, Anna Kydd, Sai Bhagra, Jason M Tarkin

**Affiliations:** Section of Cardiorespiratory Medicine, University of Cambridge, Victor Phillip Dahdaleh Heart and Lung Research Institute, Papworth Road, Cambridge Biomedical Campus, Cambridge, CB2 0BB, UK; Department of Radiology, University of Cambridge, Cambridge, UK; Department of Radiology, Royal Papworth Hospital, Cambridge, UK; Department of Radiology, Royal Papworth Hospital, Cambridge, UK; Department of Cardiology, Royal Papworth Hospital, Cambridge, UK; Department of Radiology, Cambridge University Hospitals NHS Trust, Cambridge, UK; Department of Radiology, Imperial College Healthcare NHS Trust, London, UK; Section of Cardiorespiratory Medicine, University of Cambridge, Victor Phillip Dahdaleh Heart and Lung Research Institute, Papworth Road, Cambridge Biomedical Campus, Cambridge, CB2 0BB, UK; Section of Cardiorespiratory Medicine, University of Cambridge, Victor Phillip Dahdaleh Heart and Lung Research Institute, Papworth Road, Cambridge Biomedical Campus, Cambridge, CB2 0BB, UK; Section of Cardiorespiratory Medicine, University of Cambridge, Victor Phillip Dahdaleh Heart and Lung Research Institute, Papworth Road, Cambridge Biomedical Campus, Cambridge, CB2 0BB, UK; Departments of Biomedical Sciences and Medicine, Cedars-Sinai Medical Center, Biomedical Imaging Research Institute, Los Angeles, CA, USA; Section of Cardiorespiratory Medicine, University of Cambridge, Victor Phillip Dahdaleh Heart and Lung Research Institute, Papworth Road, Cambridge Biomedical Campus, Cambridge, CB2 0BB, UK; Section of Cardiorespiratory Medicine, University of Cambridge, Victor Phillip Dahdaleh Heart and Lung Research Institute, Papworth Road, Cambridge Biomedical Campus, Cambridge, CB2 0BB, UK; Transplant Unit, Royal Papworth Hospital, Cambridge, UK; Transplant Unit, Royal Papworth Hospital, Cambridge, UK; Section of Cardiorespiratory Medicine, University of Cambridge, Victor Phillip Dahdaleh Heart and Lung Research Institute, Papworth Road, Cambridge Biomedical Campus, Cambridge, CB2 0BB, UK

**Keywords:** heart transplantation, coronary allograft vasculopathy, coronary computed tomography angiography, pericoronary adipose tissue density

## Abstract

**Aims:**

To assess pericoronary adipose tissue (PCAT) density on coronary computed tomography angiography (CCTA) as a marker of inflammatory disease activity in coronary allograft vasculopathy (CAV).

**Methods and results:**

PCAT density, lesion volumes, and total vessel volume-to-myocardial mass ratio (V/M) were retrospectively measured in 126 CCTAs from 94 heart transplant patients [mean age 49 (SD 14.5) years, 40% female] who underwent imaging between 2010 and 2021; age- and sex-matched controls; and patients with atherosclerosis. PCAT density was higher in transplant patients with CAV [*n* = 40; −73.0 HU (SD 9.3)] than without CAV [*n* = 86; −77.9 HU (SD 8.2)], and controls [*n* = 12; −86.2 HU (SD 5.4)], *P* < 0.01 for both. Unlike patients with atherosclerotic coronary artery disease (*n* = 32), CAV lesions were predominantly non-calcified and comprised of mostly fibrous or fibrofatty tissue. V/M was lower in patients with CAV than without [32.4 mm^3^/g (SD 9.7) vs. 41.4 mm^3^/g (SD 12.3), *P* < 0.0001]. PCAT density and V/M improved the ability to predict CAV from area under the receiver operating characteristic curve (AUC) 0.75–0.85 when added to donor age and donor hypertension status (*P* < 0.0001). PCAT density above −66 HU was associated with a greater incidence of all-cause mortality {odds ratio [OR] 18.0 [95% confidence interval (CI) 3.25–99.6], *P* < 0.01} and the composite endpoint of death, CAV progression, acute rejection, and coronary revascularization [OR 7.47 (95% CI 1.8–31.6), *P* = 0.01] over 5.3 (SD 2.1) years.

**Conclusion:**

Heart transplant patients with CAV have higher PCAT density and lower V/M than those without. Increased PCAT density is associated with adverse clinical outcomes. These CCTA metrics could be useful for the diagnosis and monitoring of CAV severity.

## Graphical Abstract

**Graphical Abstract:**
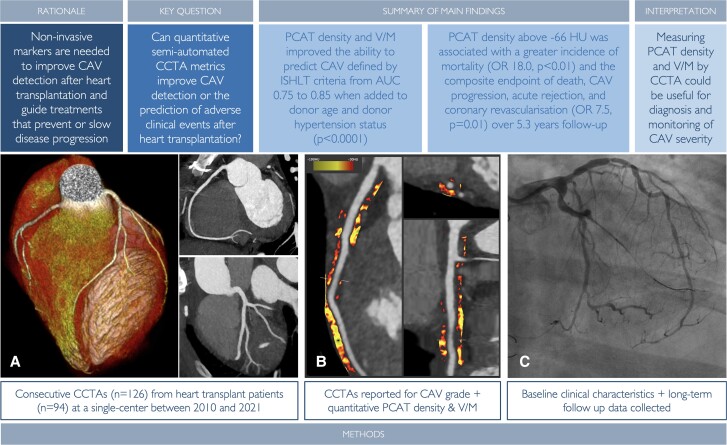
A schematic overview of the main study aims and findings, with representative images showing (*A*) CCTA reconstructions in 3D and multi-planar views, along with corresponding (*B*) color map demonstrating increased baseline PCAT density in the left anterior descending artery of a heart transplant patient with grade 1 CAV at the time of their CCTA, who subsequently died of allograft rejection. (*C*) X-ray angiographic images of another heart transplant patient with multifocal CAV lesions requiring percutaneous revascularization (n.b. coronary guidewire *in situ*). AUC, area under the receiver operating characteristic curve; CAV, coronary allograft vasculopathy; CCTA, cardiac computed tomography angiography; ISHLT, international society for heart and lung transplantation; OR, odds ratio; PCAT, pericoronary adipose tissue; V/M, vessel volume-to-myocardial mass ratio.

## Introduction

Coronary allograft vasculopathy (CAV) is the leading cause of late-transplant mortality in patients who survive the first year after heart transplant.^1^ CAV is an accelerated form of coronary disease characterized by diffuse, concentric intimal hyperplasia associated with vascular inflammation caused by mononuclear cell infiltration and T cell responses.^2^ Depending on the imaging modality used, CAV is observed in 42–75% of all cardiac transplants within 3 years of transplantation.^3^ Treatments for CAV include statins, immunosuppressive agents, and percutaneous or surgical revascularization. However, often these treatments fail to prevent the need for re-transplantation in patients with advanced CAV who develop severe heart failure; hence, methods for early CAV detection are needed to guide treatments that can slow or prevent disease progression.^4^

Identifying CAV at an early stage in the disease is challenging because CAV can develop at any time after transplantation, often in the absence of clinical symptoms. While invasive angiography ± intravascular imaging are the most accurate methods of diagnosing CAV, angiography alone cannot detect mild intimal thickening in early CAV, and intravascular imaging catheters are too large to pass into small, distal vessels that are often the first to be affected and often the most severely.^5,6^ Coronary computed tomography angiography (CCTA) is an alternative imaging modality for CAV, which is non-invasive and, unlike stress imaging modalities, has the advantage of being able to detect mild, non-flow limiting lesions.^7,8^ CCTA can also be used to assess coronary inflammation by measuring pericoronary adipose tissue (PCAT) density,^9,10^ which could provide an important marker of disease activity in CAV. Moreover, the ratio of coronary artery volume-to-myocardial mass (V/M) on CCTA^11^ could also be useful for identifying diffuse, distal tapering (or ‘pruning’) in patients with CAV that can be difficult to distinguish by conventional angiography.

We tested the hypotheses that (i) PCAT density and/or V/M quantified by CCTA could improve the ability to diagnose CAV in heart transplant patients, and (ii) these CCTA metrics would be associated with adverse clinical outcomes.

## Methods

### Study design and participants

In this retrospective observational cohort study, consecutive CCTAs were analysed from cardiac transplant patients who underwent imaging as part of usual clinical care at the Royal Papworth Hospital, Cambridge, UK, between 2010 and 2021. CCTAs from heart transplant patients were also compared with age, sex, and tube voltage–matched CCTAs of patients with normal coronary arteries and patients with atherosclerotic coronary artery disease (CAD), performed as part of clinical care. External ethics committee review was not required for this retrospective analysis, as confirmed by The Royal Papworth Hospital Department of Research and Development.

### Imaging

CCTA was performed using either a single- or dual-source scanner [64-slice SOMATOM Definition (scans performed before 2014) or 192-slice SOMATOM Force (scans performed after 2014), Siemens Healthcare, Germany] following a standard clinical protocol. Prior to imaging, patients received sublingual glyceryl trinitrate (400 µg) ± intravenous metoprolol as needed to achieve a steady heart rate below 65 bpm, provided there was no contraindication. Following a test bolus to determine the delay from time of contrast injection to image acquisition, intravenous contrast (Omnipaque 350 mg/mL, GE Healthcare, Milwaukee, WI, USA) was administered at 5 mL/s followed by a 0.9% saline flush. Electrocardiogram-gated CCTA images were acquired either prospectively or retrospectively, during end-inspiration with the patients’ arms positioned above their heads. Tube current (smart mA) and voltage (smart kV, 70–120) were adjusted for body size and reconstructed at 0.75 mm slice thickness.

### Image analysis

CCTAs were assessed for CAV and graded according to the International Society for Heart and Lung Transplant (ISHLT) scoring system by two experienced radiologists as per clinical care, independent of the research study.^12^ Anonymized CCTAs were analysed by a single reader who was blinded to the ISHLT grade and other clinical details using validated and reproducible methods (Autoplaque Version 2.5, Cedars Sinai Medical Center).^13^ This analysis included semi-automated CCTA vessel segmentation, quantitative plaque analysis, and measurement of average PCAT density around epicardial coronary artery segments with diameter ≥2 mm. Total, calcified, non-calcified, and low-attenuation [<30 Hounsfield units (HU)] plaque volumes were calculated and used to derive respective measures of %plaque burden (plaque volume × 100/vessel volume). PCAT density around each coronary segment was determined automatically as the mean attenuation in HU of voxels with tissue ranging from −190 up to −30 HU within a predefined volume of interest located in a 3 mm radius of the outer coronary wall, which was free of contamination from the myocardium or coronary arterial branches. Mean PCAT density in each patient was calculated for all coronary segments (mPCAT_total_) and in proximal (mPCAT_prox_), mid/distal (mPCAT_mid/distal_), and proximal right coronary artery (mPCAT_pRCA_) segments. For consistency, mPCAT_total_ was chosen for all subsequent analyses. Although PCAT density measured in the proximal right coronary artery is a commonly employed method for atherosclerosis research, we focused on mPCAT_total_ in this study because the coronary distribution of CAV tends to be more diffuse. Myocardial mass was measured by semi-automated segmentation software (Syngovia, Siemens Healthcare, Germany) and used to generate total vessel volume-to-myocardial ratio (V/M). Myocardial attenuation was measured in the basal inferoseptum.

### Statistical analysis

Continuous variables were expressed as mean [standard deviation (SD) or standard error of the mean (SEM)] as appropriate. Patient-level CCTA metrics, demographic, and clinical data were compared between groups using the unpaired Student’s *t*-test and/or Pearson’s product moment coefficient. Linear mixed-effects modelling was used to account for hierarchical data as some patients contributed more than one scan to the analysis, with the statistical significance assessed using Satterthwaite’s degrees of freedom method. Diagnostic accuracy was assessed by receiver operating characteristic analysis, with thresholds selected by Youden’s index. Multivariable regression analysis included all potential confounding variables with *P* < 0.1 in the Student’s *t*-test and univariable analysis. Statistically significant variables were modelled as predictors in a generalized linear model to assess their accuracy for discriminating CCTAs with CAV from those without. The association of PCAT density and V/M with all-cause mortality and the predefined composite endpoint of death, CAV progression (defined as increased CAV grade assessed by CCTA, invasive coronary angiography, or ischaemia testing), acute allograft rejection (biopsy confirmed), and coronary revascularization was compared using an odds ratio (OR) for the risk of an event occurring during the study period. A two-sided *P* < 0.05 was considered significant. Statistical analyses were performed using R statistical analysis program R (Version 4.1.0, R Core Team). Violin plots were generated using Prism (Version 9.1.0, GraphPad Software).

## Results

### Clinical characteristics

**Figure 1:**
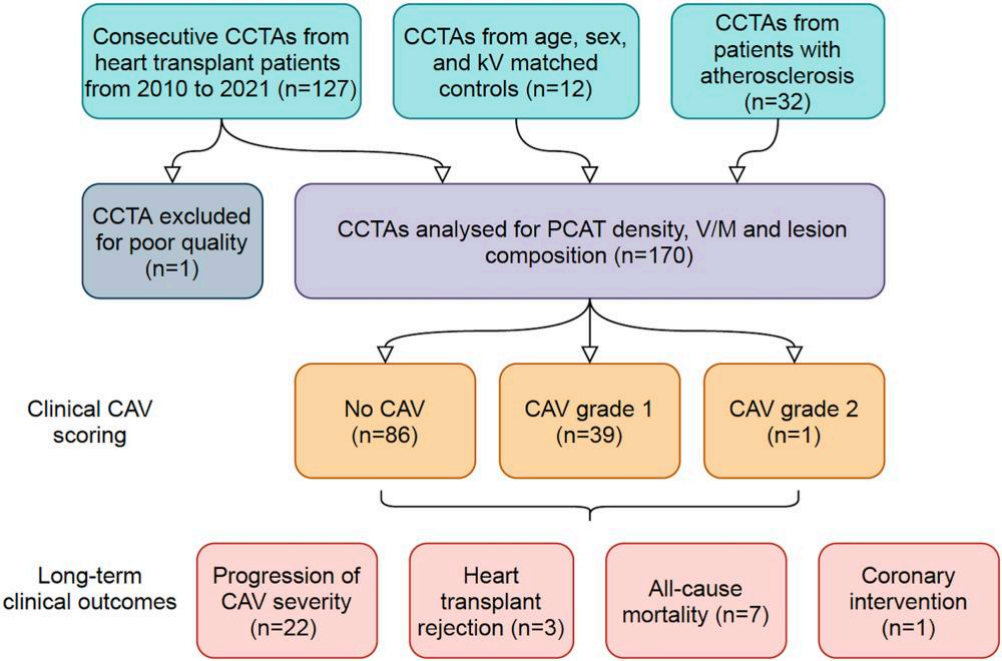
Study flowchart.

A total of 126 consecutive CCTAs were analysed from 94 patients who were imaged after cardiac transplantation during the study period (*Figure 1*). Fifteen scans were performed on a SOMATOM Definition scanner, and the other 111 scans were performed on the same SOMATOM Force scanner. Baseline patient demographics for scans with CAV (*n* = 40) and without CAV (*n* = 86) are shown in *Table 1*. There was a mean 968 (SD 802) days between transplant and CCTA for the whole cohort. Biochemical markers of vascular inflammation were sought, but results that were contemporaneous with CCTA were not available for most patients. Of CCTAs with CAV grade >0, 39/40 (97.5%) had CAV grade 1 and 1/40 (2.5%) had CAV grade 2. One scan was excluded from the analysis due to poor image quality. A similar number of patients had a history of early transplant rejection prior to CCTA in both groups. There were no patients with coronary stents. Associations between CAV grade and clinical risk factors are shown in *Table 2*.

**Table 1:** Baseline characteristics

	CAV grade = 0	CAV grade ≥1	*P*-value
**CCTA parameters**			
CCTAs analysed, *n*	86	40	
Patient age (SD) at time of CCTA	50.7 (13.7)	46.4 (15.9)	0.14
Days (SD) between transplant and CCTA	989 (805)	931 (797)	0.65
CCTAs that were follow-up scans, *n* (%)	23 (26.7%)	9 (22.5%)	
Total coronary segments analysed, *n* (SD)	795	364	
Coronary segments per CCTA, *n* (SD)	9.2 (0.85)	9.1 (1.1)	1
Mean tube voltage, kV (SD)	88.4 (15.7)	93.3 (15.4)	0.10
Mean tube current, mA (SD)	1614 (509)	1357 (543)	0.01
**Recipient parameters**			
Age at transplantation, years (SD)	48.57 (13.7)	44.24 (15.6)	0.14
Sex (% female)	52 (61)	23 (58)	0.68
Height, cm (SD)	173 (8.9)	171 (9.2)	0.35
Weight, kg (SD)	76.6 (15.2)	72.9 (11.5)	0.13
Body mass index, m/kg^2^ (SD)	25.6 (4.3)	24.9 (3.3)	0.29
Smoking history, *n* (%)	30 (37.5)	14 (35.8)	0.88
Diabetes mellitus, *n* (%)	8 (9.0)	1 (3.0)	0.13
Hypertension, *n* (%)	10 (12.3)	1 (2.5)	0.03
CMV positive, *n* (%)	43 (51)	17 (42)	0.36
Rejection within 12 months, *n* (%)	28 (33)	19 (48)	0.12
**Donor parameters**			
Donation after brainstem death, *n* (%)	74 (86)	35 (88)	0.77
Donor ischaemia time, min (SD)	176 (55.8)	184 (73.6)	0.52
Sex (% female)	50 (58)	27 (68)	0.30
Age, years (SD)	35.2 (10.9)	44.4 (9.2)	<0.001
Height, cm (SD)	175 (9.0)	172 (98.4)	0.15
Weight, kg (SD)	78 (16.4)	83 (16.0)	0.07
Smoking history, *n* (%)	0.55 (50)	0.55 (50)	0.99
Hypertension, *n* (%)	20 (24)	26 (65)	0.01
Diabetes mellitus, *n* (%)	1 (1.0)	3 (7.0)	0.15
CMV positive, *n* (%)	33 (38)	16 (40)	0.86
CMV mismatch, *n* (%)	68 (80)	25 (63)	0.27

CCTA, coronary computed tomography angiogram; CMV, cytomegalovirus.

**Table 2:** Correlation between risk factors and CAV grade

	CAV grade 0	CAV grade ≥1	Univariable correlation with CAV grade	Univariable *P*-value	Multivariable *P*-value
Donor age^a^, years (SD)	35.2 (9.2)	44.4 (10.9)	0.37	<0.0001	0.0001
Donor hypertension^a^, *n* (%)	20.0 (24)	26.0 (65)	0.22	0.01	0.09
mPCAT_total_ density, HU (SD)	−77.9 (8.3)	−73.0 (9.3)	0.25	0.004	0.0002
mPCAT_prox_, HU (SD)	−75.0 (9.6)	−70.9 (10.6)	0.19	0.03	0.003
mPCAT_mid/distal_, HU (SD)	−80.0 (8.03)	−74.6 (9.0)	0.29	0.001	0.0001
mPCAT_pRCA_ density, HU (SD)	−79.2 (11.7)	−75.0 (11.8)	0.24	0.004	0.01
Myocardial attenuation, mean HU (SD)	138.8 (43.1)	123.8 (34.3)	−0.17	0.04	0.25
Total vessel V/M, mm^3^/g (SD)	41.4 (12.3)	32.4 (9.6)	−0.35	<0.0001	0.002

HU, Hounsfield units; mPCAT, mean pericoronary adipose tissue; V/M, total vessel volume-to-myocardial mass ratio.

^a^Clinical factors identified as potential confounding factors from *Table 1*; recipient hypertension not included as there was an inverse relationship not in keeping with known clinical observations.

### Association of PCAT density and CAV grade

**Figure 2:**
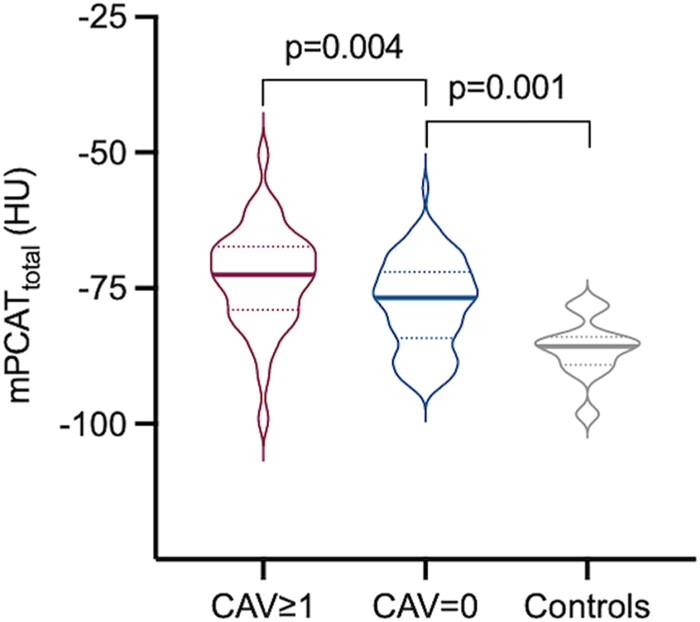
PCAT density in patients with and without CAV. Violin plots showing PCAT (mPCAT_total_) density in CCTAs from patients grouped by CAV status and age-, sex-, and kV-matched controls with normal CCTAs and no heart transplant. Data expressed as median (interquartile range).

PCAT density was increased in heart transplant patients with CAV compared to those without CAV [mPCAT_total_: −73.0 HU (SD 9.3) vs. −77.9 HU (SD 8.2), *P* = 0.004; mPCAT_pRCA_: −75 HU (SD 11.8) vs. −79.2 HU (SD 11.7), *P* = 0.006; mPCAT_mid/distal_: −74.6 HU (SD 8.0) vs. −80.0 HU (SD 9.0), *P* = 0.001; *Table 2* and *Figure 2*]. Age, sex, and tube voltage–matched CCTAs from patients with normal coronary arteries and no heart transplant [*n* = 12; mean age 54.5 (SD 13.2); 58% (7/12) female; mean kV 93.33 (SD 15.6)] were analysed as controls. mPCAT_total_ density in the healthy control subjects [−86.2 HU (SD 5.4)] was lower than in heart transplant patients, regardless of CAV grade (*P* < 0.0001; *Figure 2*). In the one patient with CAV grade 2, PCAT density was numerically higher than the other patients with CAV grade 1 at −69.8 HU. As all the PCAT metrics performed similarly, mPCAT_total_ was chosen for further multivariable analysis and modelling for consistency and because of the diffuse nature of the disease.

These findings were confirmed in the linear mixed model, which showed a 4.76 ± SEM 1.60 difference between PCAT values from heart transplant patients with CAV compared to those without CAV (*P* = 0.001) and an 11.99 ± SEM 3.7 difference in PCAT between heart transplant patients with CAV and healthy controls (*P* < 0.0001). The model of CT scanner was also considered in the mixed linear regression model and was not found to be a significant contributor to the relationship between PCAT and CAV grade. Although 32 heart transplant patients had repeat CCTAs during the study period, only 3 patients had progression of CAV grade based on CCTA criteria; hence, it was not possible to examine the association between change in PCAT density or V/M on CTCA and CAV progression.

### Other factors associated with CAV grade

Donor age, donor hypertension, and V/M were also associated with CAV grade (*Table 2*). Notably, V/M was reduced in heart transplant patients with CAV compared to those without [32.4 mm^3^/g (SD 9.64) vs. 41.4 mm^3^/g (SD 12.3), *P* < 0.0001]. These factors were included in the multivariable predictive model for CAV, along with PCAT density. Although recipient hypertension was also identified as a potential predictor, a weak inverse relationship was found that is out of keeping with known clinical observations and likely insignificant. As the inclusion of recipient hypertension had very minimal impact on the overall results, this factor was not included in the final model. Myocardial attenuation was also correlated with CAV grade on univariable analysis, but this factor was not significant when it was applied in the multivariable model. Similarly, although mA and donor weight had *P* < 0.1 for difference between the groups, these factors were not significant in the multivariable model. There was no difference in kV between the groups, which is known to affect HU. There was no association between V/M and time from transplant to CCTA (*P* = 0.60).

### Integrative model for predicting CAV grade

**Figure 3:**
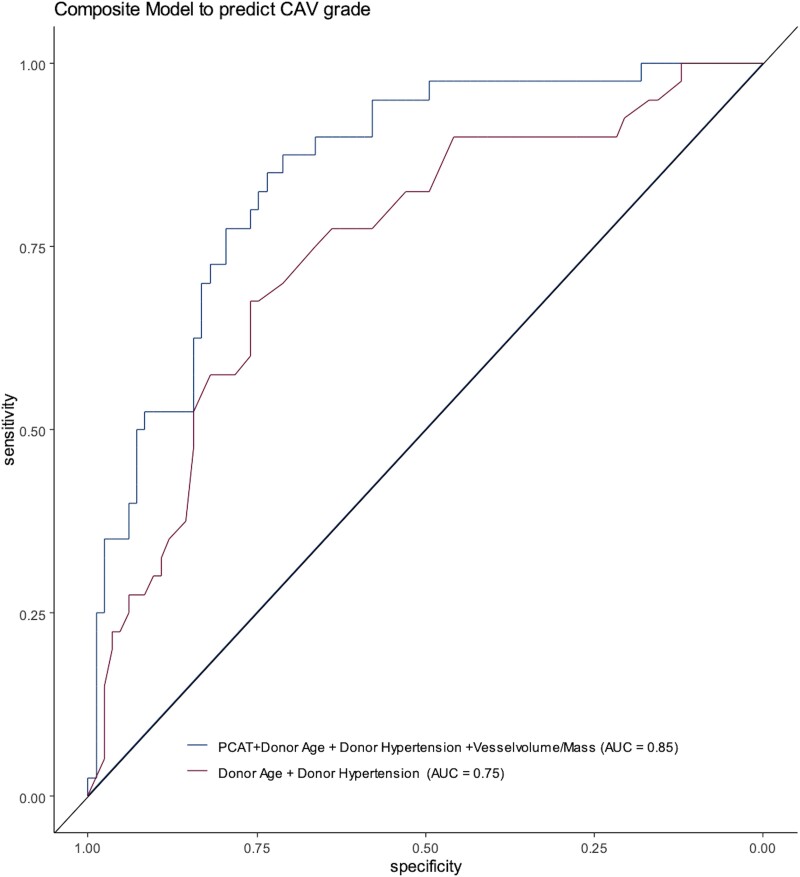
Diagnostic accuracy of clinical and CCTA predictors of CAV. Receiver operating characteristic curve showing the added value of PCAT density and V/M to clinical factors for predicting CAV.

Clinical and imaging factors associated with CAV grade were considered together in a generalized linear model to assess their combined ability to distinguish CCTAs with CAV grade ≥1 from those CAV grade = 0. These factors included donor age, donor hypertension, mPCAT_total_ density, and V/M. The overall adjusted Akaike information criterion (AIC) values for the clinical (two-factor) and clinical + CCTA (four-factor) models were 138.8 (*P* < 0.0001) and 119.8 (*P* < 0.0001) with a ΔAIC of 20 and a mean standard error of 18.1 and 14.7%, respectively. The sensitivities, specificities, and diagnostic accuracies for the clinical and clinical + CCTA models are shown in *Table 3* and *Figure 3*. Overall, the addition of mPCAT_total_ and V/M to age and donor hypertension status improved the diagnostic accuracy from area under the receiver operating characteristic curve (AUC) 0.75 to AUC 0.85 (*P* < 0.0001). The clinical + CCTA model demonstrated a sensitivity of 78% [95% confidence interval (CI) 65–90] and specificity of 80% (95% CI 71–88) for differentiating CCTAs with CAV grade ≥1 from CAV grade = 0.

**Table 3:** Diagnostic accuracy of predictors for CAV grade ≥1

	Sensitivity	Specificity	AUC
Clinical predictors: donor age + donor hypertension	71.1 (95% CI 61.5–80.7)	70.0 (95% CI 55.0–82.5)	0.75
CCTA predictors: mPCAT_total_ density + V/M ratio	68 (95% CI 52.5.0–80.0)	67 (95% CI 55.8–76.7)	0.75
Clinical + CCTA: donor age, donor hypertension, PCAT density, and V/M	78 (95% CI 65–90)	80 (95% CI 71–88)	0.85

HU, Hounsfield units; PCAT, pericoronary adipose tissue; V/M, total vessel volume-to-myocardial mass ratio.

### Quantitative CCTA characterization of CAV lesions

Quantitative lesion analysis was compared in heart transplant patients with CAV (*n* = 40) to those with stable atherosclerotic CAD and no heart transplant (*n* = 32). Patients with atherosclerotic CAD had clinical demographics reflective of the underlying disease, with a mean age of 65 (SD 8.92) years and 19% (6/32) were female. CAV lesion composition in patients was almost exclusively non-calcified (99.9% non-calcified plaque burden), predominantly fibrotic and fibrofatty, with very minimal calcification based on established thresholds for discriminating tissue density with CCTA (see Supplementary data online, *Table S1*). In comparison, patients with atherosclerotic CAD had a higher proportion of calcification and necrotic core as would be expected due to the differences in underlying disease pathologies. PCAT density was weakly associated with total plaque burden (*r* = 0.17, *P* < 0.0001) and non-calcified plaque volume (*r* = 0.17, *P* < 0.0001) across all patients with heart transplant or atherosclerotic CAD (see Supplementary data online, *Table S3*). When restricted to only patients with heart transplant, fibrous plaque volume was the only association that remained (*r* = 0.12, *P* < 0.0001). Again, as only 3 of 32 patients who underwent follow-up CCTA developed progression of CAV based on CCTA criteria after the baseline scan, it was not possible to statistically compare apparent increases in plaque volumes observed in these patients to those without CAV progression on repeat CCTA.

### Comparison of clinical factors, PCAT density, and V/M with clinical outcomes

**Figure 4:**
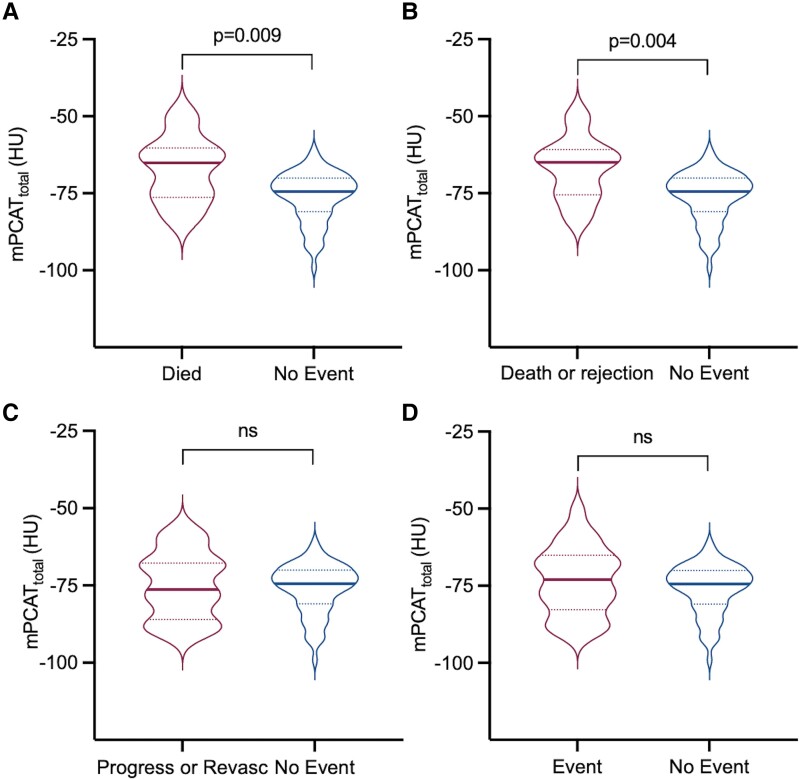
PCAT density in patients with and without adverse clinical outcomes. Violin plots of PCAT (mPCAT_total_) density in patients who (*A*) died vs. no event; (*B*) death or rejection vs. no clinical event; (*C*) CAV progression or revascularization vs. no event; and (*D*) any clinical event vs. no clinical event. Data expressed as median (interquartile range). ns = non-significant.

In the 94 patients with heart transplant who underwent CCTA in this study, clinical outcomes were assessed over a mean 5.3 (SD 2.1)-year period. No patients were lost to follow-up. There were 7 deaths, of which 2 (29%) were due to allograft rejection. A total of 27 patients had an adverse clinical event included in the composite endpoint (see Supplementary data online, *Table S2*). Of the 21 patients who had CAV progression, 14 were graded as CAV 0 and 7 were CAV 1 at baseline CCTA. Baseline mPCAT_total_ density was higher in patients who died compared to those who had no event during the follow-up period [−67.14 HU (SD 10.92) vs. −76.04 HU (SD 8.06), *P* = 0.009]. There was also a difference in mPCAT_total_ for death or transplant rejection vs. no clinical events [−66.89 HU (SD 10.15) vs. −76.04 HU (SD 8.06), *P* = 0.004], but not for CAV progression, revascularization, or the composite endpoint of all adverse clinical outcomes (*Figure 4*). Of note, one of the three patients with acute transplant rejection also had a previous history of rejection before their baseline CCTA, but overall, there was no association between early rejection and mPCAT_total_ density (*P* = 0.29). None of the clinical factors associated with CAV grade (donor age and donor hypertension) or other baseline data listed in *Table 1* were associated with a greater incidence of death, transplant rejection, or the composite endpoint (*P* > 0.1 for all). There was also no association between V/M and future adverse events.

**Figure 5:**
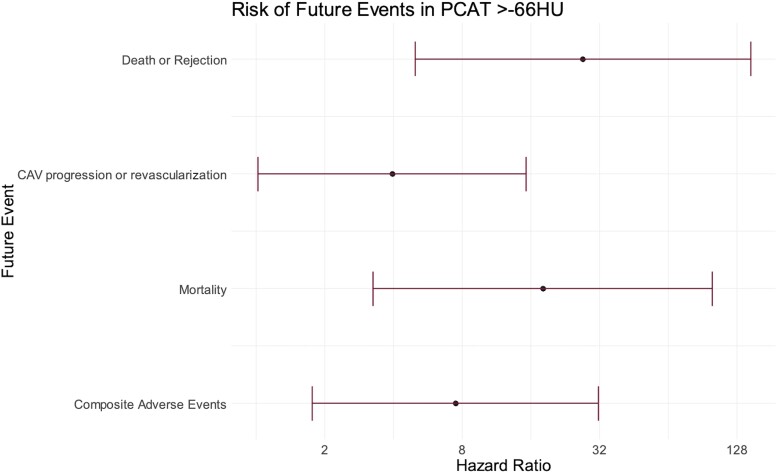
Association of very high PCAT density with adverse clinical outcomes. Forrest plot demonstrating the elevated risk of individual and composite adverse events in patients with PCAT (mPCAT_total_) density above −66 HU.

In patients with very high PCAT density, the association with clinical events was even more apparent. mPCAT_total_ above −66 HU was selected as the optimal threshold based on the Youden index. Patients with mPCAT_total_ above −66 HU had an increased incidence of all-cause mortality [OR 18.0 (95% CI 3.25–99.6), *P* = 0.002] and the composite endpoint of death, CAV progression, acute rejection, and coronary revascularization [OR 7.47 (95% CI 1.76–31.6), *P* = 0.01] over the study period (*Figure 5*). In total, 4/10 (40%) of patients with a PCAT density above −66 HU died compared to 3/84 (3.6%) with PCAT density below −66 HU. For allograft rejection, all 3 patients with biopsy-proven rejection had a PCAT density >−66 HU (3/10 patients with a PCAT >−66 HU, compared to 0/84 patients with a PCAT <−66 HU). Similarly, 7/10 (70%) of patients with a PCAT density above −66 HU had an event included in the composite endpoint, compared to 20/84 (26.2%) of those with a PCAT density below −66 HU. Anecdotally, the only patient in the study to die of acute transplant rejection had the highest PCAT density on baseline CCTA of any patient in the study (*Graphical Abstract*).

## Discussion

Diagnosing CAV in patients with heart transplantation is challenging. Individuals are often asymptomatic in the early stages of the disease, and CAV can develop at any time post-transplant, meaning that regular screening is required. CAV can also be difficult to distinguish from atherosclerosis, which is either pre-existing in the donor heart or subsequently develops. CCTA is an attractive non-invasive imaging option for diagnosis and monitoring of CAV. Here, for the first time, we showed that PCAT density and V/M could improve the diagnostic accuracy of CCTA for CAV, and that increased PCAT density was a strong predictor of adverse clinical outcomes in a large, single-centre cohort of patients with heart transplant.

### Current challenges for CAV detection and management

Although the risk of developing CAV after heart transplantation is known to be associated with several clinical risk factors,^14^ the condition remains largely unpredictable. CCTA is increasingly used for CAV screening and surveillance based on the current ISHLT grading system. However, this grading system does not consider disease activity status and may be less well suited for detecting early stages of the disease before the onset of significant luminal stenosis. Immunosuppressive medications that can slow CAV progression are most effective if initiated early, but are not without risks of side effects.^15^ Hence, there is a need both to improve early CAV detection and to identify patients with active disease who require more aggressive therapies.

### Evaluation of quantitative CCTA metrics for identifying CAV

In this study, we evaluated the use of PCAT density and V/M for improving CAV detection. PCAT density has been developed and validated as a marker of coronary inflammation in atherosclerosis but could also be of use in other vascular inflammatory diseases.^9,10^ As this marker has not previously been tested in CAV, we evaluated several different PCAT metrics to determine which method was best suited for the disease. Across all metrics tested (mPCAT_total_, mPCAT_prox_, mPCAT_mid/distal_, and mPCAT_pRCA_), we found a consistently strong association with CAV grade. In addition, we tested V/M as a novel quantitative CCTA metric for refining the diagnosis in CAV. This marker was also strongly associated with the presence of CAV on CCTA. When added to clinical risk factors, PCAT density and V/M led to an incremental improvement above clinical factors (AUC 0.87 vs. 0.75) in the ability to predict the presence of CAV as defined by conventional CCTA evaluation. Lastly, we compared CCTA-defined lesion composition in patients with CAV and atherosclerotic CAD as this distinction can be important for guiding management. We found that, unlike atherosclerosis, CAV lesions are predominately non-calcified. This finding is in keeping with other studies of CCTA imaging in patients with CAV,^16,17^ as well as histological studies.^18^ Unlike in atherosclerotic CAD, only fibrous plaque volume was correlated with PCAT density, which is again consistent with the underlying disease pathology.^19^

### Association of PCAT density and adverse clinical outcomes in CAV

Previous studies have shown associations between PCAT density and the risk of major adverse clinical events in atherosclerotic CAD.^9,20^ In our study, heart transplant patients with the highest PCAT density also had the greatest incidence of adverse clinical events. PCAT density above −66 HU was associated with a markedly increased incidence of both all-cause mortality (18-fold greater odds) and the composite endpoint of death, CAV progression, acute rejection, and coronary revascularization (7.5-fold greater odds) over a mean follow-up of 5.3 years. The latter observation was driven predominately by death and allograft rejection as only one patient in the study underwent coronary revascularization. These findings suggest that coronary inflammation assessed by PCAT density could represent an important prognostic marker in patients with CAV. While it is possible that PCAT density is also altered by myocardial inflammation in heart transplant patients,^21,22^ myocardial attenuation was not associated with CAV severity in the statistical model. However, the lack of association could also be reflective of the limitations of detecting subtle myocardial abnormalities by measuring attenuation on CT, which does not account for various factors including the timing of acquisition during the wash-in phase and grade of interstitial fibrosis related to patient-specific factors. The lack of association between early rejection before baseline CCTA and mPCAT_total_ density also does not support myocardial inflammation as an important confounding factor. Unlike PCAT density, there was also no association between baseline V/M on CCTA and subsequent clinical outcomes. The inability of this anatomical marker to predict future clinical events highlights the need to consider additional markers of disease activity. In the future, technological advances such as photon-counting CT technology with higher spatial resolution and spectral imaging could help to further delineate PCAT density in small distal coronary branches <2 mm that are known to be affected in CAV.

### Monitoring of CAV progression

In addition to the use of semi-automated quantitative CCTA metrics for detecting active CAV at an early stage in the disease when treatments may be most effective, these markers could also potentially improve the ability to track disease progression over time. This is because visual grading of CAV on CCTA is susceptible to inter-observer variability, and the pattern of CAV associated with distal pruning can be difficult to identify without comparison to previous scans. Only three patients who underwent serial CCTA scanning in this study had progression of CAV, meaning that we were not able to formally evaluate PCAT density and V/M for this use. For most of the 22% of patients with CAV progression, this change was detected by invasive angiography or stress imaging. Whether the addition of these novel parameters to standard CCTA reporting can improve early CAV detection and/or the ability to track disease progression and responses to therapy are important topics for future research.

### Study limitations

As a retrospective single-centre observational study, there are several limitations to acknowledge that could affect the generalizability of our findings. Firstly, only individuals who underwent CCTA imaging after heart transplant were included, and so patients referred directly for invasive angiography or other imaging tests have not been captured. It is possible that these patients undergoing other forms of imaging besides CCTA had a higher clinical suspicion of obstructive coronary disease. This could in part explain why nearly all patients with CAV had grade 1 (mild) disease at the time of CCTA, and why only one patient in the study required coronary revascularization during the follow-up period. As the cohort consisted of patients with mostly mild CAV or without CAV, it was also not possible to assess whether more severe CAV grades are associated with higher PCAT density. Although a degree of CAV progression occurred in almost a quarter of patients, serial monitoring of CAV severity in these individuals tended not to be with CCTA, which limited the ability to measure temporal changes in CCTA metrics in these patients. Lastly, due to the retrospective nature of the study, contemporaneous blood markers of inflammation were not available to compare with CT findings, but this would be interesting for future research. PCAT density has previously been shown to be weakly associated with several serum inflammatory biomarkers in atherosclerotic CAD.^23^

## Conclusion

Measurement of PCAT density and V/M by CCTA could be useful to identify and risk stratify individuals who develop CAV after heart transplant. While both PCAT density and V/M added incremental value as novel diagnostic markers in this study, information about inflammatory disease activity was more strongly associated with longer-term clinical outcomes than anatomical disease severity. Future studies are needed to assess the clinical utility of these semi-automated quantitative CCTA markers for tracking disease progression and response to therapy in patients with CAV.

## Supplementary data

Supplementary data are available at *European Heart Journal - Cardiovascular Imaging* online.

## Supplementary Material

jeae069_Supplementary_Data

## Data Availability

Data will be made available upon request after publication of the primary research manuscript.
