# Supplementary material for: Computed tomography pericoronary adipose tissue density predicts coronary allograft vasculopathy and adverse clinical outcomes after cardiac transplantation
Source: Eur Heart J Cardiovasc Imaging. 2024 Mar 17;25(7):1018–27. doi: 10.1093/ehjci/jeae069 (PMC11210971; doi:10.1093/ehjci/jeae069)
Supplement: jeae069_Supplementary_Data [file jeae069_supplementary_data.docx]

**Supplemental Material**

**CT pericoronary adipose tissue density predicts coronary allograft vasculopathy and adverse clinical outcomes after cardiac transplantation**

Christopher Wall,^1^ Jonathan Weir-McCall,^2,3^ Katharine Tweed,^3^ Stephen P Hoole,^4^ Deepa Gopalan,^5,6^ Yuan Huang,^1^ Andrej Corovic,^1^ Marta Peverelli,^1^ Damini Dey,^7^ Martin R Bennett,^1^ James HF Rudd,^1^ Anna Kydd,^8^ Sai Bhagra,^8^ Jason M Tarkin^1^

^1^Section of Cardiorespiratory Medicine, University of Cambridge, Cambridge, UK

^2^Department of Radiology, University of Cambridge, Cambridge, UK

^3^Department of Radiology, Royal Papworth Hospital, Cambridge, UK

^4^Department of Cardiology, Royal Papworth Hospital, Cambridge, UK

^5^Department of Radiology, Cambridge University Hospitals NHS Trust, Cambridge, UK

^6^Department of Radiology, Imperial College Healthcare NHS Trust, London, UK

^7^Departments of Biomedical Sciences and Medicine, Cedars-Sinai Medical Center, Biomedical Imaging Research Institute, Los Angeles, California

^8^Transplant Unit, Royal Papworth Hospital, Cambridge, UK

**Supplemental Table 1 | CCTA lesion composition in CAV vs. atherosclerosis**

|  | **CAV** | **Atherosclerotic CAD** | **p-value** |
| --- | --- | --- | --- |
| CCTAs analysed, n | 40 | 32 | - |
| Coronary segments analysed, n | 364 | 305 | - |
| %Fibrous plaque, mean (SD) | 26.5 (17.8) | 18.8 (15.7) | <0.001 |
| %Necrotic core, mean (SD) | 16.3 (9.5) | 26 (25.5) | <0.001 |
| %Dense calcific plaque, mean (SD) | 0.0 (3.5) | 11.4 (17.4) | <0.001 |
| %Non-calcific plaque, mean (SD) | 99.6 (2.3) | 91.5 (13.3) | <0.001 |
| %Fibrofatty plaque, mean (SD) | 55.9 (12.6) | 42.3 (20.5) | <0.001 |

*CAD: coronary artery disease; CAV: coronary allograft vasculopathy; CCTA: coronary computed tomography angiography*

**Supplemental Table 2 | Summary of clinical outcomes**

|  | **PCAT density above -66 HU** | **PCAT density below -66 HU** | **Odds Ratio** | **p-value (chi-squared)** |
| --- | --- | --- | --- | --- |
| Total patients, n (%) | 10 | 84 | - | - |
| All-cause mortality, n (%) | 4 (40) | 3 (4) | 18 | <0.001 |
| Composite endpoint of adverse clinical events (death, CAV progression, transplant rejection, or coronary intervention), n (%) | 7(70) | 22(26) | 7.47 | 0.002 |
| CAV progression, n (%) | 5(50) | 17(20) | 3.94 | 0.04 |
| Allograft rejection, n (%) | 3(30) | 0(0) | - | - |
| Coronary intervention, n (%) | 0(0) | 1(1) | - | - |

*CAV: coronary allograft vasculopathy; HU: Hounsfield Units; PCAT: pericoronary adipose tissue density*

**Supplemental Table 3| Relationship between PCAT density and lesion composition**

|  | **Heart transplant PCAT density** | **P value** | **Heart transplant + atherosclerotic CAD PCAT density** | **P value** |
| --- | --- | --- | --- | --- |
| Total Plaque Burden (%) | 0.03 | 0.2 | 0.17 | <0.0001 |
| Non-Calcific Plaque Burden (%) | -0.01 | 0.7 | 0.17 | <0.0001 |
| Total Plaque Volume (mm3) | 0.01 | 0.67 | 0.08 | 0.002 |
| Fibrous Plaque Volume (mm3) | 0.12 | <0.0001 | 0.18 | <0.001 |
| Fibro Fatty Volume (mm3) | 0.0 | 0.99 | 0.1 | 0.0002 |
